# Perception and adaptation of receptive prosody in autistic adolescents

**DOI:** 10.1038/s41598-024-66569-x

**Published:** 2024-07-16

**Authors:** Chigusa Kurumada, Rachel Rivera, Paul Allen, Loisa Bennetto

**Affiliations:** 1 Brain and Cognitive Sciences, University of Rochester, Rochester, 14627 USA; 2 Psychology, University of Rochester, Rochester, 14627 USA; 3 Otolaryngology, University of Rochester Medical Center, Rochester, 14642 USA

**Keywords:** Human behaviour, Language, Perception

## Abstract

A fundamental aspect of language processing is inferring others’ minds from subtle variations in speech. The same word or sentence can often convey different meanings depending on its tempo, timing, and intonation–features often referred to as prosody. Although autistic children and adults are known to experience difficulty in making such inferences, the science remains unclear as to why. We hypothesize that detail-oriented perception in autism may interfere with the inference process if it lacks the **adaptivity** required to cope with the variability ubiquitous in human speech. Using a novel prosodic continuum that shifts the sentence meaning gradiently from a statement (e.g., “It’s raining”) to a question (e.g., “It’s raining?”), we have investigated the perception and adaptation of receptive prosody in autistic adolescents and two groups of non-autistic controls. Autistic adolescents showed attenuated adaptivity in categorizing prosody, whereas they were equivalent to controls in terms of discrimination accuracy. Combined with recent findings in segmental (e.g., phoneme) recognition, the current results provide the basis for an emerging research framework for attenuated flexibility and reduced influence of contextual feedback as a possible source of deficits that hinder linguistic and social communication in autism.

## Introduction

Spoken communication goes beyond literal meanings of words; it is about understanding what the speaker meant to say. From subtle variations in the rhythm, timing, and intonation of speech—known as speech prosody—we routinely process a vast amount of linguistic, social, and affective meaning^1–4^. Atypicality in the production of prosody has long been associated with individuals with autism spectrum disorder^5–10^. In contrast, the ability to comprehend prosody (i.e., receptive prosody) in autism is much less well understood, and the evidence is mixed. Using prosodic contours associated with contrasting meaning, some have reported largely intact discrimination or categorization performance in autism^3,11–14^. Others, however, found significant impairments or mixed results across tasks^9,12,15–19^. In addition, population heterogeneity as well as near-ceiling performance seen in some tasks have limited definitive interpretation of the data^9,20^.

Critically, work on receptive prosody in autism thus far has overlooked a fundamental function of human speech processing: the accommodation of speech from multiple different speakers. In everyday speech, different speakers produce the same prosodic category (e.g., question vs. statement intonation) in physically different ways^21,22^. This ambiguity is readily apparent when considering multiple social and dialect groups (e.g., a speaking style often referred to as “uptalk” in which a non-question has a rising final pitch^23^). Moreover, there are numerous individual differences that lead to widespread ambiguity in prosodic meaning in natural language^24^. For example, Xie et al. (2021) elicited 3,000+ tokens of questions and statements from 65 monolingual native speakers of American English from a campus community in the northeastern United States^25^. Even within this relatively homogeneous population, one speaker’s question can have a pitch contour indistinguishable from another speaker’s statement (or vice versa).

This is consistent with the critical idea in the broader speech science literature that robust speech processing must involve “a balancing act”^26^. To comprehend prosody, listeners must be exquisitely sensitive to prosodic cues (e.g., fundamental frequency (F0), timing, duration) to avoid confusing categories. Yet, at the same time, they must be flexible enough to adapt to variable inputs, such as those produced by speakers of different ages, genders, physiological characteristics, and social and geographic dialects^27–31^. Failure to adapt, as shown in a computational simulation by Xie et al. (2021), significantly reduces the accuracy of recognizing the intonational meaning. In short, for robust receptive prosody, it is not enough to achieve accurate perception; listeners must also be able to accommodate the variability in the cue-meaning mapping across speakers.

In this study, we investigated the accuracy and adaptivity of receptive prosody in autistic adolescents (aged 13–17 years). Many autistic adolescents without verbal impairments are as adept as their neurotypical peers in categorizing the basic, grammatical meaning of prosody (e.g., question vs. statement)^9,32^. However, their ability to cope with the prosodic variability is currently unknown. Addressing this question may help to link linguistic and social factors in language comprehension in autism during adolescence. Recent evidence suggests that neurotypical children and younger adolescents show a greater degree of brain response to socially and biologically more salient (e.g., mother’s) voices than to unfamiliar voices; in contrast, older adolescents tend to show the opposite tendency, with increased activity to unfamiliar voices over familiar voices^33^. This preference for a familiar voice at earlier stages appears to be weaker in autistic children, possibly contributing to atypical social and linguistic development^34–37^. Here we compare autistic and non-autistic adolescents, as well as a control group of young adults, to assess their ability to accommodate, and adapt to, the prosodic productions of an unfamiliar speaker.

**Figure 1:**
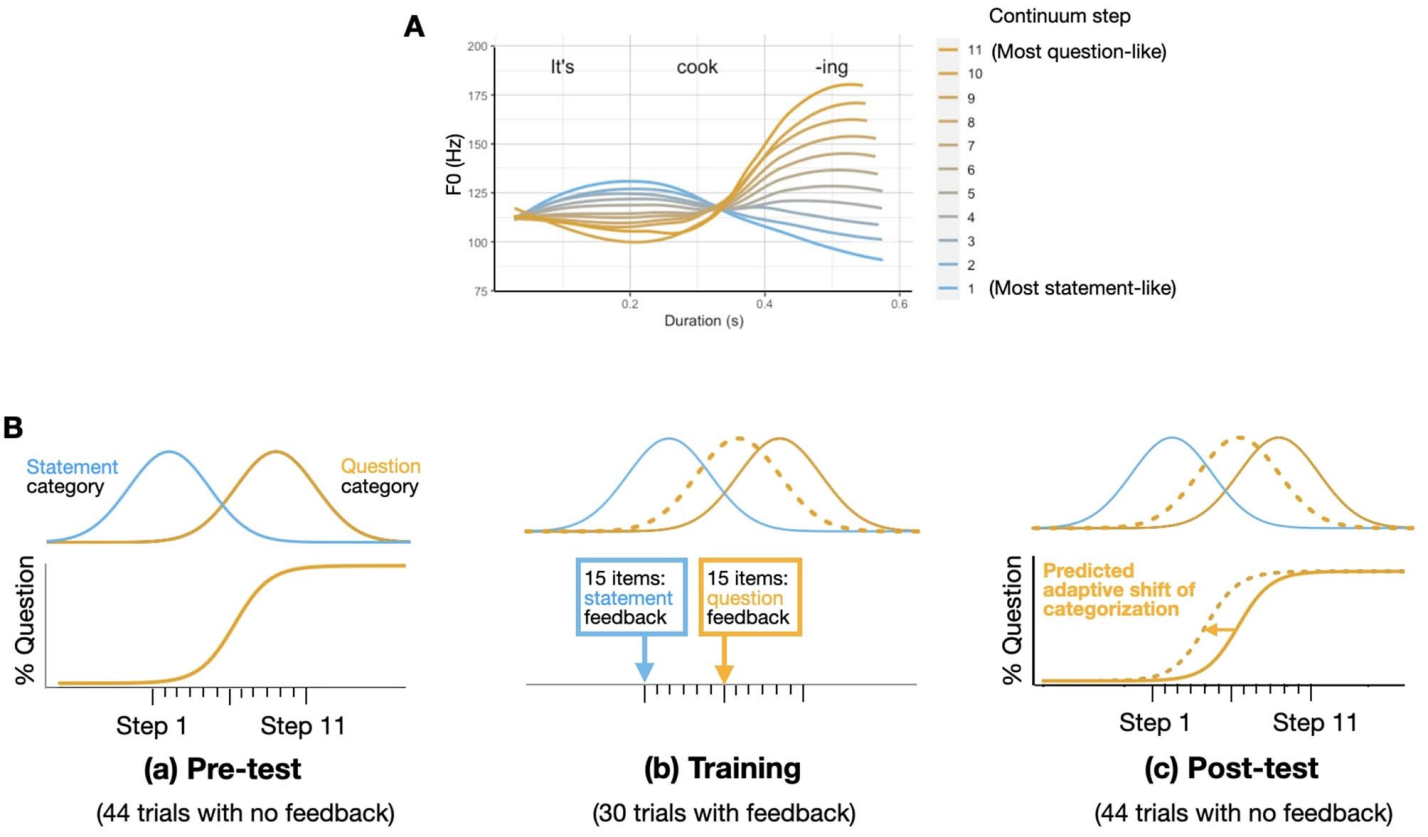
Stimuli and procedure of the adaptation task. (**A**) The 11-step gradient continuum. (**B**) The pre-test, training, and post-test design. In (**a**) pre-test, participants respond to 44 trials of 2AFC questions (“Is this person telling you something or asking you a question?”). They indicate their categorization responses by clicking on a “.” icon for statement and a “?” icon for question. The stimuli are uniformly sampled along the 11-step continuum and presented in a randomized order. In (**b**) training, participants responded to 30 trials of 2AFC questions in the same format and received feedback after each trial. 50% of the trials were accompanied by “statement” feedback and the rest were accompanied by “question” feedback. Finally, in (**c**) the post-test, participants repeated the same procedure as in (**a**). The dashed lines show the expected shift of the underlying category distributions and the resulting (predicted) shift of the categorization function.

To this end, we used the paradigm often referred to as “perceptual recalibration”. This paradigm was developed originally to test the adaptivity of speech perception at the segmental (e.g., phoneme) level^38–40^. In our experiment, participants were exposed to gradient continuum between a prototypical question and a prototypical statement (Fig. 1A). During training, they heard tokens midway between the two ends of the continuum followed by feedback disambiguating the intended meaning (e.g., “That’s right. He was asking you something”) (Fig. 1B). By comparing responses before and after this training, we determined whether their categorization responses had adapted to the prosody of the current speaker. In addition, we used the same continuum to administer a perceptual discrimination task. By departing from the use of categorical stimuli, our paradigm allows for greater sensitivity to between-group and between-participant differences in receptive prosody, in terms of both accuracy and adaptivity in response to variable prosodic productions.

## Methods

All procedures were approved by the Research Subjects Review Board of the University of Rochester, and the study was performed in accordance with all relevant guidelines and regulations. Adult participants provided informed consent and adolescent participants provided assent following parental permission.

## Participants

57 autistic and 53 non-autistic adolescents (ages 13–17 years) as well as 53 non-autistic young adults (ages 18–24 years) completed this experiment. All participants were monolingual, native speakers of American English. Autistic adolescents were recruited through the SFARI Base SPARK database (Simons Foundation Autism Research Initiative, https://www.sfari.org/). At the request of the research team, SFARI Base research liaisons prescreened participants according to our inclusion criteria. Inclusion criteria for the autistic group included (a) a previous professional diagnosis of autism, confirmed via parent report on the Social Responsiveness Scale (SRS-2^41^) (a standardized measure of autism symptomatology), (b) no known hearing/vision problems or intellectual disabilities, and (c) communicates in fluent speech. Additionally, although a greater proportion of the autistic population is male, we specifically sought to recruit a balanced gender ratio.

Non-autistic (NA) adolescents were recruited through the University of Rochester Clinical and Translational Science Institute (UR CTSI) Research Participant Registry as well as school districts in the Northeastern U.S. The inclusion criteria for this group were (a) no previous professional diagnosis of autism, confirmed by a score on the SRS-2 below the autism cutoff, and (b) no known hearing/vision problems or intellectual disabilities. In both groups, a parent or guardian of the participant responded to a demographic background and language questionnaire and the SRS-2.

In addition, we included a control group of NA young adults from the University of Rochester undergraduate population. As noted above, the processes involved in prosodic perception likely undergo developmental changes during adolescence. The increased preference for unfamiliar voices and the increased diversity of social and linguistic input during adolescence may facilitate adaptation to unfamiliar speech patterns. The inclusion of the young adult group serves as a reference point against which the adolescents’ performance can be assessed. Inclusion criteria for this group included (a) no known current or past diagnosis or suspected diagnosis of autism, and (b) no hearing/vision problems.

We excluded participants who did not reach the predetermined criterion of 75% on suprathreshold trials in the perceptual discrimination task (see Procedure and Results for more detail). Table 1 presents the demographic and background information of the participants who were included in the final analysis sample. The autistic and NA adolescents were matched by group on chronological age (*t*(98) = 0.82, *p* = 0.41) and gender composition (proportion of male participants, *χ*^2^ (1, *n* = 100) = 1.03, *p* = 0.31).

**Table 1:** Demographic information and measures of participants included in the final data analysis sample.

Group	Autistic adolescents	NA adolescents	NA young adults
N	50	50	50
Mean age in years (SD)	15.5 (1.33)	15.2 (1.45)	20.3
Female/Male/Nonbinary	17/32/1	20/27/3	24/25/1
Professional diagnosis of autism	100%	0%	0%
Mean SRS-2 Total T-score (SD)	75.4 (1.2)	43.9 (4.9)	N/A

## Stimuli

### Prosodic adaptation task stimuli: the 11-step continuum

The stimuli were adopted from a previously published study by Xie et al. (2021). They consisted of six continua in the form of “It’s X-ing” with six different verbs: *booting*, *cooling*, *cooking*, *losing*, *muting*, and *moving*. They were created based on natural productions of a statement (e.g., “It’s cooking.”) and a question (e.g., “It’s cooking?”) by a male native speaker of American English in his mid 20’s. The mean F0 and duration of the three syllables (It’s-X-ing) were used to resynthesize the 11-step continuum (Fig. 1A).

The recordings (two tokens per item) were first segmented into three regions corresponding to three syllables (i.e., It’s | X | ing). To achieve the best resynthesis results, the segmentation was done based on a turning point in the F0 contour within the “X-ing” part and segmental information to delineate the last two syllables. The F0 of each region was sampled at 20 equally spaced time points, and the measures from each time point were aggregated across items to derive mean F0 contours for the statement and question contours. Similarly, durations of each region were averaged across items by contour type. Mean F0 contours and durations were then derived by interpolating between values within each region and manipulating the F0 and duration of each recording to match the interpolated values using the pitch-synchronous overlap-and-add algorithm implemented in Praat^42,43^. See Kurumada et al. (2017)^44^ and Xie et al. (2021)^25^ for details.

### The perceptual discrimination task stimuli

**Figure 2:**
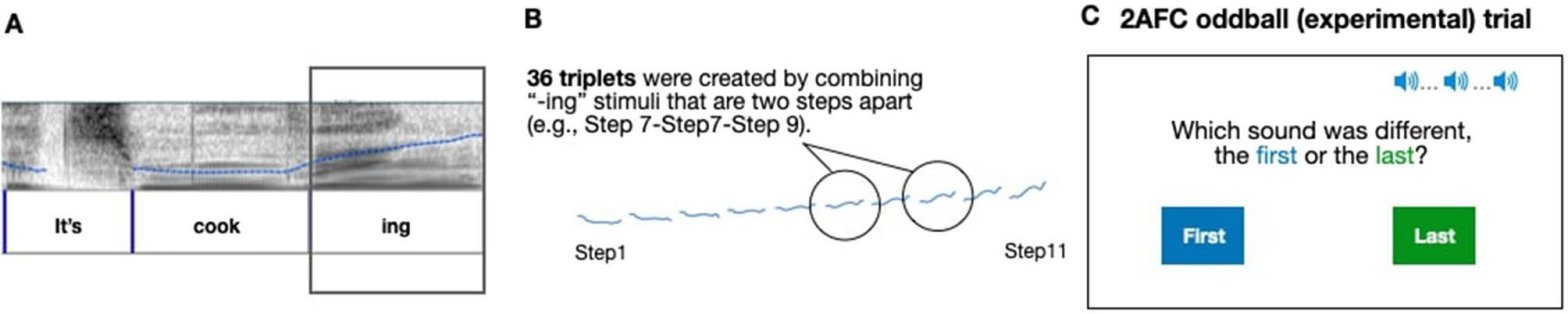
The 2AFC oddball discrimination task stimuli and procedure. (**A**) The final syllable (“-ing”) was spliced out of the 11-step test stimuli. (**B**) Nine pairs of “-ing” tokens were created by combining those that were two steps apart. Then, 36 unique triplets of the “-ing” tokens were created by crossing the oddball identity and location. (**C**) On each trial, participants heard a triplet and identified the location of the oddball stimulus by clicking the “First” or “Last” button presented on the screen. The procedure was identical between the standard and the suprathreshold trials.

The stimuli for the discrimination task were created by splicing out the final syllable from the 11-step continua used in the pre- and post-tests (i.e., The “-ing” part of “It’s cooking”, Fig. 2A). We used the spliced stimuli for three reasons. First, we chose the speech-based stimuli over other options (e.g., musical or pure tones) because of the well-known speech-specific deficits in autism^14,45–48^. To test participants’ ability to discriminate subtly different intonation contours, we chose to use the acoustically and indexically rich human speech stimuli. Second, the brevity of the “-ing” stimuli reduced the task difficulty for all participants; memorizing and judging three instances of a sentence-long intonation contour (e.g., for “It’s cooking”) would confound working memory capacity and stimulus discrimination. Finally, the task was meant to elucidate basic perceptual sensitivity, not linguistic categorization of speech sounds. The use of a content word such as “cooking” would blur the boundary between the two, as it likely evokes lexically- and exemplar-driven processes^49^. The semantic opacity of “-ing” would minimize this possibility.

The 11 steps of -ing stimuli were combined into nine pairs of items that are two steps apart from each other (e.g., Step 1-3, 2-4, 3-5, Fig. 2B). These items were then used in a three-item, two-alternative forced choice (2AFC) oddball task. Participants were presented with a triplet of these “-ing” items at a time and asked to identify the item that was different from the other two (Fig. 2C). The oddball was always played either first or last. For each pair, we created 36 unique triplets by crossing (a) nine stimuli pairs (e.g., 1-3, 2-4, 3-5), (b) two oddball identities (e.g., 1 or 3 for the 1-3 pair), and (c) two oddball locations (i.e., First or Last). Additionally, we created “suprathreshold” (very easy) stimuli that consist of items that are six steps apart. Four unique triplets were created (i.e., 3-3-9, 3-9-9, 9-3-3, 9-9-3). These stimuli were used to screen out participants who may not be able to perceive prosodic differences between the continuum steps.

## Procedure

The study was conducted online, using the platform Gorilla.sc (https://app.gorilla.sc/) for stimulus delivery^50^. Adolescent participants were tested individually by a researcher via Zoom. An experimental session consisted of three phases: (a) introduction and volume adjustment, (b) the adaptation task, and (c) the discrimination task, as we describe below. The researcher verbally explained the tasks according to a script, and the participant was invited to ask questions at any time. During the experimental trials, the researcher turned off their video and microphone, but the participant’s video/audio was left on so that the researcher could monitor for any background noise or visible signs of attentional drift. The procedure was mostly identical for NA young adults except that they completed the tasks at their own pace (i.e., without an experimenter present on Zoom).

All participants were instructed to wear a headset, earphones, or earbuds to minimize the effect of environmental noise. NA young adults who reported using external or computer speakers were excluded prior to data review. 33% of the adolescent participants chose to use speakers due to sensory sensitivities or unavailability of suitable equipment. In this case, the researcher present during the Zoom session confirmed that the environment was quiet for the duration of the tasks. Using a pre-determined set of criteria, we excluded data from participants who experienced significant background noise, other environmental distractions, or technical difficulties (Supplementary Information).

### Introduction and volume adjustment

A session began with a brief introduction and volume adjustment procedure. Participants listened to a ringing tone (450 Hz, 65 decibels) through their own listening device, and adjusted the volume setting on their own computer to a comfortable listening level.

### The prosodic adaptation task

The adaptation task consisted of three blocks: pre-test, training, and post-test (Fig. 1B). The pre- and post-test blocks each contained 44 trials (11 steps * four repetitions of “It’s cooking”). The presentation order was randomized for each participant and in each block. Participants responded to an auditory stimulus by clicking on an icon presented on the screen with the labels of “telling” or “asking”. In each trial they heard a stimulus only once and received no feedback throughout.

The training block included 30 tokens of “It’s X-ing” utterances containing five different verbs with the /u/ vowel (*moving*, *losing*, *muting*, *booting*, and *cooling*, six instances each). The task remained the same as in the test blocks, except that participants received feedback after each categorization response (e.g., “That’s right. He was telling you something”, “Actually, he was telling you something”). The feedback was accompanied by a visual icon of a happy or sad face indicating the correct or incorrect answer, respectively, with a high-pitched ring tone (= correct) or a low-pitched buzzer (= incorrect). Each participant heard 15 tokens of unambiguous statements, with feedback indicating the statement meaning, and 15 tokens of ambiguous utterances, with feedback indicating that the token was a question. Participants never heard the prototypical question in this block.

Previous online testing with NA adults^25^ has demonstrated that this manipulation induces an overall increase in “question” responses in the post-test, indicating an adaptive shift in prosody categorization. By manipulating the feedback, the same study also has induced an opposite shift with increased “statement” responses. However, the magnitude of the shift was smaller. Given the expected heterogeneity of responses in the autistic population, we chose the current question-biasing manipulation because of its robust expected effect. Additionally, the current design was motivated by a previous study^19^, in which autistic adolescents exhibited a strong tendency to judge a declarative question as a statement. The current “question-biasing” manipulation, we reasoned, would serve as a stronger test of adaptation than the “statement-biasing” one, which would simply reinforce this bias.

### The perceptual discrimination task

Each participant responded to 80 trials (72 standard stimuli and eight suprathreshold stimuli, presented in a randomized order). In each trial, they listened to a triplet stimulus and indicated an oddball location by clicking on a printed word on the screen (“First” or “Last”, Fig. 2C). A demonstration and four practice trials, with feedback and an opportunity to replay stimuli, were given to ensure the task comprehension. To reduce fatigue, we divided the task into two blocks of equal length with a short break in between.

To ensure task engagement, the discrimination trials were interspersed with four attention-check trials. Three simple shapes appeared on the screen, and participants were asked to click on one of them according to a printed instruction (e.g., “Click on the red star to continue.”). Performance on these trials helped identify participants who did not follow instructions and/or were prone to attentional lapses. We used a predetermined 75% accuracy cutoff for screening to focus our analysis on those with sufficient levels of attention and task engagement.

## Predictions

For the perceptual discrimination task, we predicted no group difference after screening for attentional checks and supra-threashold trials. For the adaptation task, we predicted a group-by-block (pre vs. post) interaction, suggesting that autistic adolescents would show attenuated adaptivity in prosodic categorization as compared to NA controls. All analyses of main effects and interactions were conducted using a mixed-effects logistic regression model (lme4 package^51^) in the statistical language R^52^. We provide a specific fixed and random effect structure for each task below.

## Results

In the current study, the discrimination task served as a control measure to account for possible differences in perceptual accuracy between groups. In addition, performances on the suprathreshold trials were used to screen out those who may not be able to perceive prosodic differences between steps. Below we report first the experimental results of the discrimination task and then those of the adaptation task.

## The discrimination task

As expected, most participants reached the predetermined cutoff accuracy of 75% on the suprathreshold trials: autistic adolescents, 92.0% (SD = 16.6), NA adolescents, 94.2% (SD = 11.5), and NA young adults 94.3% (SD = 11.6). 13 participants who did not meet this threshold were excluded from further analysis. These included 7/57 (12.2%) in the autistic adolescent group, 3/53 (5.7%) in the NA adolescent group, and 3/53 (5.7%) in the NA young adult group; Fisher’s exact test (p= .44) indicates that the exclusion rate did not differ significantly between groups.

**Figure 3:**
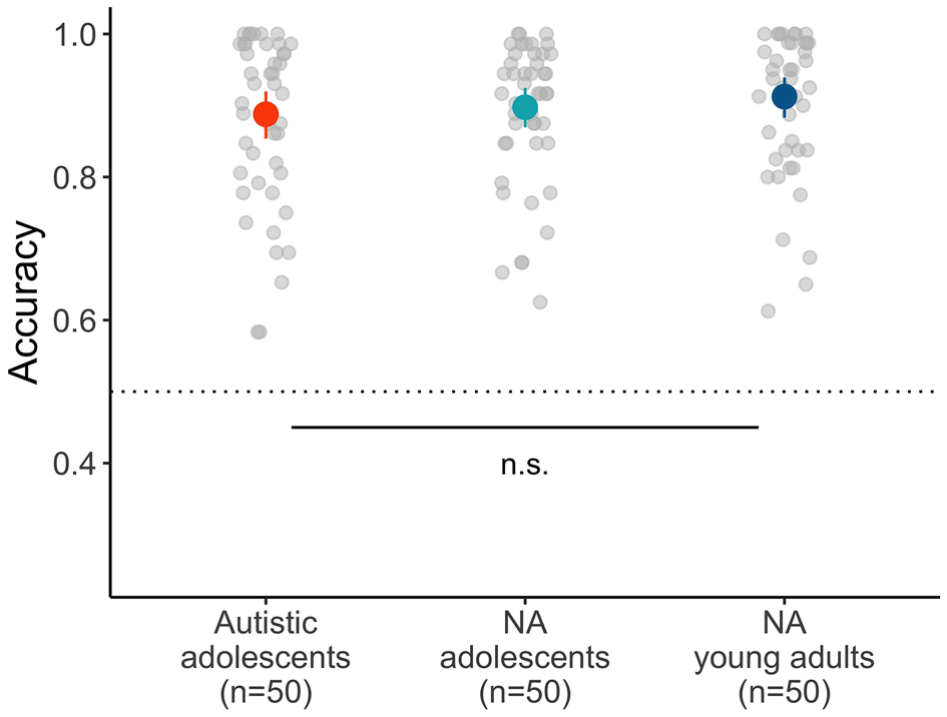
Mean accuracy of responses to the standard trials in the discrimination task by group. Error bars indicate bootstrapped 95% CIs. The gray dots in the background represent the individual subject’s mean accuracy. Data shown here include only participants who exceeded the 75% accuracy threshold on the suprathreshold trials.

The response accuracy on the standard trials for the final sample (after exclusions) is depicted in Fig. 3. The three groups clearly exceeded the chance performance of 50% (autistic adolescents, 88.8% (SD = 11.7), NA adolescents, 89.7% (SD = 9.8), and NA young adults 91.2% (SD = 10.1)).

The analysis focused only on responses to the standard trials. We predicted listeners’ trial-level response accuracy (1 = correct vs. 0 = incorrect) based on one fixed effect variable: group (factorial, treatment-coded with autistic adolescents as the reference level). This coding allows us to compare the response accuracy of the two control groups against that of the autistic adolescent group. We considered the maximal random effect structure justified by the design (Barr et al., 2013): participant and item (e.g., a pair of steps such as Step 1 and Step 3). The final model included random intercepts for participant and item, as well as by-item slopes for group.

Table 2 provides the model summary. There was no significant effect of group (NA adolescents vs. autistic adolescents, ($\widehat{\beta }$= 0.049, *z* = 0.159); NA young adults vs. autistic adolescents, ($\widehat{\beta }$ = 0.391, *z* = 1.24)), suggesting that the three groups were equally accurate in the discrimination of subtly varying prosodic stimuli.

**Table 2:** Fixed-effect estimates of the generalized linear mixed-effect model predicting the discrimination accuracy by group.

	Estimate	StdError	z	p-value
(Intercept)	2.654	0.220	12.054	< 2e–16***
NA adolecents (vs*.* Autistic adolescents)	0.049	0.314	0.159	0.874
NA young adults (vs*.* Autistic adolescents)	0.391	0.315	1.24	0.215

## The adaptation task

**Figure 4:**
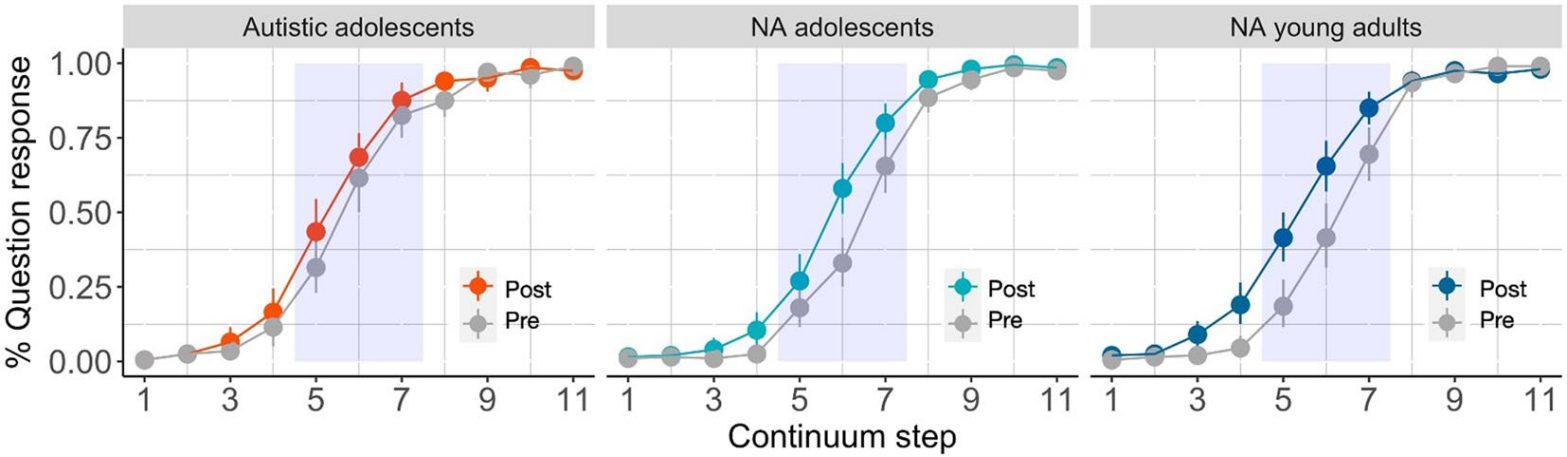
Proportions of responses to questions during pre- (gray) and post-test (colored) by group. Error bars indicate bootstrapped 95% CIs. The light blue box in the background is added to each panel for visual inspection. It shows the mid-range (steps 5–7) of the continuum where the shift in response from pre-test to post-test is expected to be most apparent. Note, however, that the statistical analysis included data points from all steps.

Figure 4 shows the proportions of question responses in the pre- and post-test blocks. Inspection of the responses supports two observations. First, steps 1 and 11 were reliably identified as statements and questions, respectively, in all groups. This means that when the contours were clearly falling or rising, the prosody-meaning mapping was easily recognizable to all, including the group of autistic adolescents. This confirms the validity of the task and the manipulation. Second, and importantly, the categorization function showed a predicted left-ward (up-ward) shift after training in the two NA control groups. On the other hand, the two categorization functions almost overlap in the autistic adolescent group.

As in the discrimination task, we used a generalized linear mixed-effect model, with family “binomial” and the logit-link function. We considered three fixed effect variables and their interaction terms: group (factorial, treatment-coded with autistic adolescents as the reference level), block (factorial, contrast-coded, post-test = 1 vs. pre-test = −1), and continuum (numerical variable of 1–11, centered). The centering of the continuum steps ensures that its interaction with the other two fixed effects is assessed at the center of the continuum step where the adaptation effect is expected to be most prominent. The final model included by-participant random intercepts and slopes for continuum.

Table 3 provides the model summary. There was a significant effect of group, such that the autistic adolescents were more likely to respond “question” overall, as compared to both NA adolescents ($\widehat{\beta }$ = − 1.10, *z* =  − 3.74, *p* = 0.0002) and NA young adults ($\widehat{\beta }$ = − 0.624, *z* =  − 2.127, *p* = 0*.*03). The expected effects of block ($\widehat{\beta }$ = 0.256, *z* = 4.282, *p* < 0*.*0001) and continuum ($\widehat{\beta }$= 1.584, *z* = 16.945, *p* < 0*.*0001) were also significant. That is, participants were more likely to respond “question” after the training than before, and in response to steps from the higher end of the continuum.

**Table 3:** Fixed-effect estimates of the generalized linear mixed-effect model predicting the question response by group, block, and continuum.

	Estimate	StdError	z	p-value
Intercept	0.964	0.213	4.523	6.08e–06***
NA adolescents (vs*.* Autistic adolescents)	− 1.10	0.294	− 3.74	0.0002***
NA young adults (vs*.* Autistic adolescents)	− 0.624	0.293	− 2.127	0.03*
Post-test (vs*.* Pre-test)	0.256	0.06	4.282	1.85e–05***
Continuum	1.584	0.093	16.945	< 2e–16***
NA adolescents * Post-test	**0.236**	**0.085**	**2.778**	0.005**
NA young adults * Post-test	**0.294**	**0.085**	**3.458**	0.0005***
NA adolescents * Continuum	− 0.058	0.125	− 0.46	0.645
NA young adults * Continuum	− 0.077	0.125	− 0.618	0.537
Post-test * Continuum	− 0.017	0.032	− 0.539	0.59
NA adolescents * Post-test * Continuum	0.01	0.047	0.222	0.824
NA young adults * Post-test * Continuum	− 0.138	0.047	− 2.95	0.003**

Key interactions are highlighted in bold.

Most critically, the group-by-block interactions were both significant: autistic adolescents vs. NA adolescents ($\widehat{\beta }$= 0.236, *z* = 2.778, *p* = 0*.*005) and vs. NA young adults ($\widehat{\beta }$= 0.294, *z* = 3.458, *p* = 0*.*0005). These interactions support our hypothesis. The adaptation effect was significantly larger in the two control groups than in the autistic adolescents. The (negative) three-way interaction term between group (NA young adults vs. autistic adolescents), block, and continuum ($\widehat{\beta }$ = − 0.138, *z* = − 2.95, *p* = 0*.*003) indicates that the group difference was even more pronounced towards the lower end of the continuum, while it was capped by the ceiling at the upper end. Taken together, the current results provide the first evidence of attenuated receptive prosody adaptation in autistic adolescents.

## Discussion

Everyday speech sounds are highly variable, both within and across speakers. The accuracy of the comprehension system must therefore be matched by its adaptivity. We found that the categorization responses of autistic adolescents differed critically from controls in their adaptivity. While their overall categorization function was very similar to that of the non-autistic groups, it was significantly less malleable before and after training.

This new finding may help to reconcile the mixed results in the literature. The intact categorization reported in some studies may be due to the use of unambiguous stimuli, where a categorical contrast in prosody (e.g., clearly rising vs. falling contours) reliably maps onto binary meanings (e.g., question vs. statement). If we had used only endpoint stimuli (Steps 1 and 11), we would also have found no difference between the three groups. Across-group differences became apparent for mid-range stimuli whose categorization depends on statistics unique to the current speaker. The slower or attenuated adaptation may mean that autistic adolescents are likely to struggle more with these ambiguous or unexpected prosodic inputs than both non-autistic peers and adults. In the future, a closer look at the nature and variability of the speech input surrounding autistic children and adolescents will prove fruitful. Such data would shed light on the nature of their expectations and where (i.e., with what type of prosodic input) they may experience comprehension difficulties.

The attenuated adaptivity in autism has been observed in a number of sensory-perceptual and decision-making tasks, such as visual^53–56^, auditory^57,58^, and tactile^59^ perception, multisensory integration^60,61^, and higher-level cognitive processing^62,63^(but see^64^). In the area of speech perception, Alispahic at al. (2022) tested autistic and neurotypical adults on the perception of fricative categories in Australian English (e.g., /f/-/s/)^65^. Consistent with our current results, their perceptual recalibration experiment revealed intact categorization of unambiguous instances of phonemes but reduced adaptation in response to exposure.

From these seemingly disparate bodies of work, a unified explanation of the perceptual, cognitive, and social features of autism is beginning to emerge. Influential theories of perceptual decision-making (e.g., a Bayesian or predictive-coding theory) suggest that we process sensory input by evaluating it against an internal world model built upon prior experiences. Robust perception is achieved by predicting the input according to this model, and by optimizing and adapting the model to the current environment. If this optimization interface is impaired, as has been suggested for autism, the world would appear highly unpredictable^66,67^ (but see^68^). Binur et al. (2022) summarize that many of the known symptoms of autism can be coherently explained by this account of attenuated or aberrant adaptation^69^. These include difficulties in dealing with uncertainty^70–73^ and the generation of rigid, repetitive behaviors that could restore predictability to perceptual experiences^67^.

Of the various perceptual and cognitive processes, speech comprehension is particularly vulnerable to loss of adaptivity. Not only do speakers differ in physiological and socio-linguistic characteristics (e.g., voice pitch, speech rate, regional or social accents), they also differ in ways to encode their meaning in language. For example, some may rely heavily on prosody to distinguish a question from a statement, while others may use syntax to do so (e.g., “It’s raining?” vs. “Is it raining?”). Moreover, prosodic productions often vary according to their emotional states and contexts^74,75^. Every individual speaker thus represents a unique model of prosody-meaning mapping, and the understanding of *what was said* depends critically on *who said it*. From this perspective, reduced processing of social-communicative cues in autism—e.g., vocal-identity recognition^35,76,77^ or facial-recognition^78^—could interfere with effective prosody-speaker mapping and thereby limit its adaptivity.

Three mechanisms warrant further investigation of reduced intonation adaptation in autism. The first is speaker normalization, in which speech cues are processed relative to a given speaker’s baseline, standardized within and across speakers^30,79^. Normalization of vocal pitch is implicated in specialized cortical areas^80,81^, the disruption of which would render cross-speaker differences in intonation a source of noise rather than a signal that aids perception. The second is perceptual learning of speaker-specific distributional statistics^29,82^. A vital facilitator of this mechanism is the ability to store previously experienced statistics and to generalize them to the same or a similar speaker, thus eliminating the need to learn them anew each time. Autism might affect this learning per se, or the generalization of learned statistics across contexts. The third is post-perceptual inference about the perceived intonation and its meaning. Non-autistic adults have been shown to account for how reliably a given speaker uses intonation to encode a linguistic meaning^83,84^. Constancy in intonation processing likely draws on these three mechanisms, independently and in concert^85^: neural diversity can thus have multiple effects on its adaptivity.

We acknowledge that some aspects of the current design limit the generalizability of the data. The autistic adolescents we tested were all verbally fluent, and our analysis focused on those who have demonstrated accurate (i.e., above-threshold) discrimination accuracy and task engagement. The results therefore cannot speak to the broader autistic population. Future studies should extend the current approach to individuals with greater language difficulties and/or a broader range of autistic traits and characteristics (for a post-hoc exploratory analysis on the possible relationship between autism severity and adaptivity, see Supplementary Information). In addition, the lack of data from younger children and autistic adults makes it difficult to resolve two alternatives. One possibility is that attenuated adaptivity in autism is a developmental delay, a gap that will be closed with age and increasing linguistic exposure. The other is that it is a symptom of atypical development that persists across age. Longitudinal or cross-sectional studies are needed to paint a fuller picture.

The current results highlight two interesting questions for future research. First, in the prosodic adaptation task, the three groups seemed to have differed in their starting points (i.e., pre-test responses). Specifically, mid-range stimuli that were more or less ambiguous to NA young adults were question-biased in autistic adolescents and statement-biased in NA adolescents. These differences were not expected a priori, and we currently lack an explanation for them. Perhaps NA adolescents may be more familiar with uptalk, a stylistic use of rising/flat prosody with the statement meaning^23^. Autistic adolescents, on the other hand, may be relatively unaware of this social, stylistic choice; they may have instead perceived the subtle hint of rising prosody as a cue to the question meaning. Our ongoing research aims to delve into autistic and NA listeners’ interpretations of prosody in relation to the speaker’s age, gender, and other social characteristics.

Finally, unlike phoneme category adaptation, where lexical identity provides immediate feedback (e.g., “peach” vs. “beach”), prosodic meanings do not always get disambiguated even after a whole sentence is heard. To circumvent this in the current experimental context, we adopted the binary distinction between question and statement meanings with explicit feedback during training (e.g., “You’re right. He was asking a question”). In contrast, much of the feedback given in natural conversation is more implicit, especially for affective meanings. Listeners must make use of nuanced linguistic and social cues, including hedges, backchannels, and facial expressions. Attenuated adaptivity in autism may also result from the reduced corrective feedback in the prosody-meaning mapping, a possibility that our work will address in the future.

## Supplementary Information

Supplementary Information.

## Data Availability

To facilitate replications and modifications of our current tasks, we have made all stimuli, the experimental paradigm, the data, and our analysis codes downloadable from our repository: https://osf.io/zqn6m/.
