# Publisher Correction: Perception and adaptation of receptive prosody in autistic adolescents

**DOI:** 10.1038/s41598-025-96909-4

**Published:** 2025-04-22

**Authors:** Chigusa Kurumada, Rachel Rivera, Paul Allen, Loisa Bennetto

**Affiliations:** 1 Brain and Cognitive Sciences, University of Rochester, Rochester, 14627 USA; 2 Psychology, University of Rochester, Rochester, 14627 USA; 3 Otolaryngology, University of Rochester Medical Center, Rochester, 14642 USA

Correction to: *Scientific Reports* 10.1038/s41598-024-66569-x, published online 16 July 2024

The original version of this Article contained an error in Table 1, where NA young adults value for “Mean age in years (SD)” was incorrect,

“2.3 (2.59)”

now reads:

“20.3”

The original Article has been corrected.

