# Supplementary material for: Perception and adaptation of receptive prosody in autistic adolescents
Source: Sci Rep. 2024 Jul 16;14:16409. doi: 10.1038/s41598-024-66569-x (PMC11252140; doi:10.1038/s41598-024-66569-x)
Supplement: Supplementary file 1 — Supplementary Information. [file 41598_2024_66569_MOESM1_ESM.pdf]

Supplementary Information for "Perception and adaptation of receptive prosody in autistic adolescents"

S.1 Steps taken to control the data validity

Our data collection took place online. We took several steps to ensure the quality of the listening environment as well as the validity of data. Most importantly, all adolescent participants were tested via the Zoom conference platform, with a research team member present throughout the study. The researcher verbally explained each task according to a predetermined script, and the participant was invited to ask questions at any time. During the experimental trials, the researcher turned off their video and microphone, but the participant's video and audio were left on so that the researcher could monitor for any background noise or visible signs of attentional drift. Additionally, the experimenter noted any other sources of distractions or technical difficulties.

1.1 Participant exclusion due to environmental and technological issues

Eight adolescent participants experienced technical or environmental problems. We followed a predetermined, conservative set of criteria to categorize these problems. If the researcher noted the presence of environmental distractions (e.g., background noise, interruptions) or technical issues (e.g., equipment malfunction), all data collected from that participant (if any) were discarded without further review. These problems included:

a) Major technical difficulties that interrupted their participation in the study (e.g., problems with Zoom, computer, listening devices, internet connectivity). (n=4)
b) Significant interruptions due to environmental distractions and background noise (e.g., other people talking in the background). (n=4)

1.2 Participant exclusion due to task-related behavioral and attentional difficulties

Four participants were excluded for behavioral and/or attentional difficulties that significantly interfered with administration. This level of screening was conducted prior to data analysis, using a predetermined set of criteria. Because these factors were related to the participant themselves, we note research group membership in the numbers below. Exclusions included:

a) Inability to achieve the 75% cut-off on the visual attention check trials (2 autistic participants)
b) Clear evidence of not responding to the acoustic properties of the stimuli (e.g., systematic alternating responses) (1 autistic participant)
c) Subject-generated distractions (e.g., consistently talking during trials that interfered with the audibility of stimuli) (1 autistic participant)

1.3 Participant exclusion based on the suprathreshold trials in the perceptual discrimination task

We included the discrimination task as a control measure to account for possible group-level differences in sensitivity to prosodic stimuli. If groups differed in their ability to perceive subtle differences in prosodic contours, possible differences in the adaptation task could be explained by these low-level

perceptual differences rather than by their adaptivity *per se*. Interspersed in the discrimination task were eight suprathreshold (i.e., very easy) stimuli that consisted of items that were six (vs. two) steps apart on the 11-step continuum. According to our predetermined cutoff of 75% accuracy on the suprathreshold discrimination trials, we removed seven participants from the autistic adolescent group, three participants from the non-autistic (NA) adolescent group, and three participants from the NA young adult group (see Table S1).

Group	Autistic adolescents	NA adolescents	NA young adults
N	7	3	3
Mean age in years (SD)	15.9 (1.37)	14.5 (1.13)	20.7 (1.15)
Female/Male/Nonbinary	3/4/0	2/1/0	1/1/1
Mean SRS-2 Total T-score (SD)	75.1 (6.09)	42 (5.29)	N/A

Table S1. Demographic information of participants who scored below 75% accuracy on suprathreshold trials in the perceptual discrimination task.

S2. Listening devices

2.1 Headphone types and quality

The current study was conducted online, and participants used their own listening devices. Remote testing of speech perception is becoming more common (Padilla-Ortiz & Orduña-Bustamante, 2021), and our current data collection protocol has been vetted by our published work (Xie et al., 2021). To further evaluate the potential effects of listening devices on stimulus audibility, we conducted a feasibility study with 48 young adults and had them complete the perceptual discrimination task online. After completing the task, we asked participants about the type and price range of the listening devices they used for the task. As can be seen in Figure S1, performance was not strongly affected by their listening devices. This motivated our decision to allow participants to use their own devices to perform the tasks of the current study.

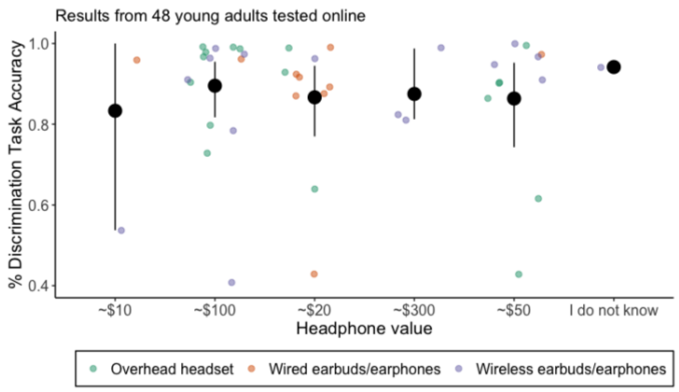

Figure S1. Feasibility test responses. 48 young adults participated in the perceptual discrimination task online. Their mean accuracies are plotted by the values of their listening devices. Error bars indicate the 95% confidence interval, and colors of points in the background indicate types of listening devices.

2.2 Listening device use during the tasks

We encouraged all participants to use external listening devices (e.g., on-/over-ear headphones, earphones, or earbuds) throughout the study. Some adolescent participants chose to use computer speakers due to sensory sensitivity or unavailability of equipment. In these cases, the presence of experimenter on Zoom ensured that the stimuli were audible throughout the experiment and that there was little or no ambient noise. Regardless of their equipment use, those who experienced significant auditory or other distractions were removed from the data (See Figure S1).

NA young adult participants were also instructed to use external listening devices. They were also asked to choose a quiet environment with no to minimal background noise and to turn off other sound sources. Because an experimenter was not present on Zoom to monitor the auditory environment during testing for this group, we applied strict criteria and removed all those who did not declare the use of external listening devices in the post-experiment questionnaire.

Group	Autistic Adolescents	NA Adolescents	NA Young Adults
Headphones/earbuds/headsets	34 (68%)	36 (72%)	50 (100%)
Computer speakers	16 (32%)	14 (28%)	0 (0%)

Table S2. Participants' listening device use by group. NA young adults' device usage was determined based on their self-report in the post-experiment questionnaire, and those not using headphones were excluded.

S.3 Post hoc exploratory analysis of autism severity and adaptivity

The current study design was optimized to achieve a comparison between the three groups of participants. Thus, it is not designed to address individual-level questions. However, it is of interest to ask whether, and if so how, the reduced adaptability found in the autistic adolescent group might be related to their autism severity. Here, we provide an initial exploratory analysis to draw implications about individual differences in the adaptation task performance. To estimate autism symptom severity, we used the total T-score derived from the Social Responsiveness Scale-2 (SRS-2) School-Aged Forms (Constantino & Gruber, 2012) for all adolescent participants. (Note that this information is not available for our young adult control participants). Higher SRS-2 T-scores indicate greater autism symptom severity.

First, we visually assessed if there was any relationship between participants' SRS-2 scores and their receptive prosody adaptation. To do so, we constructed a generalized linear mixed-effects model similar to the one reported for the adaptation task with the random by-participant intercepts and slopes for block (instead of continuum). **We then used the `coef` function from the library "stats (ver. 3.6.2)" to extract by-participant random slopes from the model results.** These provide estimates of the effect of block (pre vs. post) on the log odds of each participant's question responses (i.e., by how much their "question" responses increased after training, controlling for all other effects in the model). We then plotted these random slope estimates against their SRS-2 scores (Figure S2). If the autism severity predicts reduced adaptivity, we should see an overall negative correlation between the two.

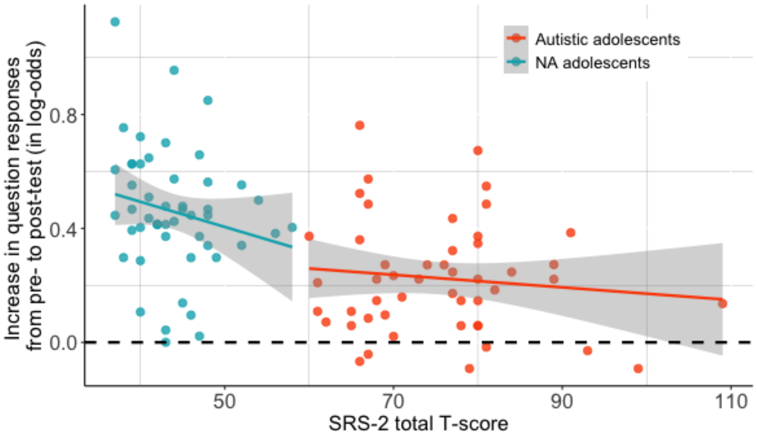

Figure S2. Increase in question responses from pre- to post-test (in log-odds), predicted in the generalized linear mixed-effects model reported in the manuscript, plotted against the SRS-2 total T-scores of adolescent participants. Points indicate individual participants, color-coded by group. The regression line was added by the function "lm" of ggplot2 and the smoother indicates the 95% confidence interval. The dashed line indicates the value of 0, indicating no difference before and after the training.

Second, we tested the effects of the categorical "group" variable and the numerical "SRS-2" variable. To do so, we built two generalized linear mixed-effect models similar to what we report on in the manuscript, with only the two adolescent groups. In one, we considered three fixed effect variables: group (factorial, contrast-coded with autistic adolescents = -1, vs. NA adolescents = 0), block (factorial, contrast-coded, post-test = 1 vs. pre-test = -1), continuum (numerical variable of 1-11, centered) as well as their interaction terms. In the other, we also included SRS-2 (numerical variable, scaled). In both cases, the final models included random intercepts by participant and random slopes for block. **We then conducted a model comparison to test whether the model with SRS-2 scores explained more variance than the model without.**

Note: "Group" and "SRS-2" are highly colinear since the two groups are categorically separated in terms of their SRS-2 scores (with the clinical cut-off score of 60). We therefore cannot draw any strong conclusion from this model comparison. This was a *post hoc*, exploratory analysis, and the results need to be interpreted with caution.

The model comparison through ANOVA suggests that the model with SRS-2 scores showed better fit to the data ($p < .0001$) (Table S3).

	npar	AIC	BIC	logLik	deviance	Chisq	df	Pr(>Chisq)
[Model without SRS-2]	11	4362.8	4440.6	-2170.4	4340.8			
[Model with SRS-2]	15	4342.5	4448.6	-2156.3	4312.5	28.21	4	1.131e-05 ***

Table S3. Summary of model comparison using ANOVA

Taken together, with all the caveats, the current data provide initial support for the idea that autism severity may predict reduced adaptivity. The SRS-2 scores appear to explain some additional variance in the data over and above the categorical-level variable of group. As can be seen in Figure S2, the higher the SRS-2 score, the smaller the adaptive effect within each group.

However, a new experimental design and additional data collection will be required to test this hypothesis more rigorously. In particular, the current results provide the following recommendations.

1) As can be seen in Figure S2, there is a substantial amount of individual variation within each group. The heterogeneity within the autistic adolescent group was expected, but the non-autistic group also showed a large amount of variability between participants. The current sample size (n=50 per group) was motivated by a previous group-level analysis (Xie et al., 2021) and is likely underpowered for individual-level analysis and prone to type II error. (Related issues are discussed in Blott et al., 2023). A larger sample would be needed for individual-level analyses.

2) In the current recruitment protocol, we used SRS-2 scores as part of our exclusion criteria. This resulted in a categorical separation of the groups in terms of their SRS-2 scores, which limits the data spread and statistical power for individual-level analysis. A wider and more diverse range of autism symptom severity—at both the autistic and non-autistic tails of the continuum—would be needed to evaluate SRS-2 effects independent of group effects.

3) Finally, as is known, an SRS-2 score does not linearly predict the autism severity. Also, it is a composite measure representing various characteristics exhibited by an individual. The linear assumption made in the models above may not be warranted and needs revising as we learn more about how autism symptomatology may affect receptive prosody comprehension.

References

Blott, L. M., Gowenlock, A. E., Kievit, R., Nation, K., & Rodd, J. M. (2023). Studying individual differences in language comprehension: The challenges of item-level variability and well-matched control conditions. *Journal of Cognition*, *6*(1). https://doi.org/10.5334/joc.317

Constantino, J. N., & Gruber, C. P. (2012). *Social Responsiveness Scale–Second Edition (SRS-2)*. Western Psychological Services.

Padilla-Ortiz, A. L., & Orduña-Bustamante, F. (2021). Binaural speech intelligibility tests conducted remotely over the Internet compared with tests under controlled laboratory conditions. *Applied Acoustics*, *172*, 107574. https://doi.org/10.1016/j.apacoust.2020.107574

Xie, X., Buxó-Lugo, A., & Kurumada, C. (2021). Encoding and decoding of meaning through structured variability in speech prosody. *Cognition*, *211*. https://doi.org/10.1016/j.cognition.2021.104619
